# Myocardial fibrosis and arrhythmic burden in systemic sclerosis

**DOI:** 10.1093/rheumatology/keac065

**Published:** 2022-02-08

**Authors:** Laura Ross, Benedict Costello, Zoe Brown, Dylan Hansen, Anniina Lindqvist, Wendy Stevens, Andrew Burns, David Prior, Mandana Nikpour, André La Gerche

**Affiliations:** Department of Medicine, The University of Melbourne at St Vincent’s Hospital; Department of Rheumatology, St Vincent’s Hospital, Fitzroy; Sports Cardiology Laboratory, Baker Heart and Diabetes Institute, Melbourne; Department of Cardiology, St Vincent’s Hospital, Fitzroy, VIC, Australia; Department of Medicine, The University of Melbourne at St Vincent’s Hospital; Department of Rheumatology, St Vincent’s Hospital, Fitzroy; Department of Rheumatology, St Vincent’s Hospital, Fitzroy; Sports Cardiology Laboratory, Baker Heart and Diabetes Institute, Melbourne; Department of Rheumatology, St Vincent’s Hospital, Fitzroy; Department of Medicine, The University of Melbourne at St Vincent’s Hospital; Department of Cardiology, St Vincent’s Hospital, Fitzroy, VIC, Australia; Department of Medicine, The University of Melbourne at St Vincent’s Hospital; Department of Cardiology, St Vincent’s Hospital, Fitzroy, VIC, Australia; Department of Medicine, The University of Melbourne at St Vincent’s Hospital; Department of Rheumatology, St Vincent’s Hospital, Fitzroy; Department of Medicine, The University of Melbourne at St Vincent’s Hospital; Sports Cardiology Laboratory, Baker Heart and Diabetes Institute, Melbourne; Department of Cardiology, St Vincent’s Hospital, Fitzroy, VIC, Australia

**Keywords:** SSc (scleroderma), myocardial fibrosis, arrhythmia

## Abstract

**Objectives:**

Cardiac complications of SSc are a leading cause of SSc-associated death. Cardiac imaging for identifying substrate abnormality may be useful in predicting risk of cardiac arrhythmias or future cardiac failure. The aim of this study was to quantify the burden of asymptomatic fibro-inflammatory myocardial disease using cardiac magnetic resonance imaging (CMR) and assess the relationship between asymptomatic myocardial fibrosis and cardiac arrhythmias in SSc.

**Methods:**

Thirty-two patients with SSc with no documented history of pulmonary vascular or heart disease underwent CMR with gadolinium and 24-h ambulatory ECG. Focal myocardial fibrosis was assessed using post-gadolinium imaging and diffuse fibro-inflammatory myocardial disease quantified using T1- and T2-mapping. CMR results were compared with an age- and sex-matched control group.

**Results:**

Post-gadolinium focal fibrosis was prevalent in SSc but not controls (30% *vs* 0%, *p* < 0.01).. T1-mapping values (as a marker of diffuse fibrosis) were greater in SSc than controls [saturated recovery single-shot acquisition (SASHA): 1584 ms *vs* 1515 ms, *P* < 0.001; shortened Modified look locker sequence (ShMOLLI): 1218 ms *vs* 1138 ms, *p* < 0.001]. More than one-fifth (22.6%) of the participants had ventricular arrhythmias on ambulatory ECG, but no associations between focal or diffuse myocardial fibrosis and arrhythmias were evident.

**Conclusion:**

In SSc patients without evidence of overt cardiac disease, a high burden of myocardial fibrosis and arrhythmias was identified. However, there was no clear association between focal or diffuse myocardial fibrosis and arrhythmias, suggesting CMR may have limited use as a screening tool to identify SSc patients at risk of future significant arrhythmias.

## Graphical Abstract

**Figure:**
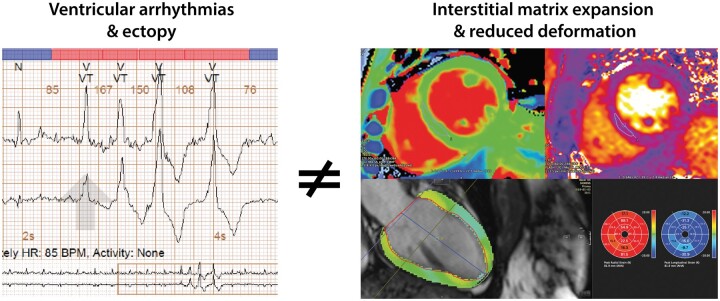
Systemic sclerosis is associated with a high prevalence of arrhythmias, exemplified by a ventricular quadruplet (left), interstitial matrix expansion and reduced longitudinal strain, exemplified by increased T1 and T2-mapping times and strain tracking (right), but there was no association between electrophysiological and imaging abnormalities detected.

Rheumatology key messages

- Fibroinflammatory myocardial disease is a near-universal finding in SSc from early in the disease course.
- Before the onset of cardiac symptoms, structural changes and electrophysiological abnormalities do not develop in parallel.
- Complete cardiac evaluation requires both structural and functional studies in addition to comprehensive rhythm interrogation.

## Introduction

Cardiopulmonary complications are the leading cause of SSc-associated death [1]. Fibrotic, inflammatory and vasculopathic changes affecting all structures of the heart have been detected in up to 80% of patients at autopsy [2]. However, clinical detection of SSc-associated heart involvement (SHI) remains challenging as findings can be subtle and non-specific [3].

It is hypothesized that cardiac arrhythmias and conduction defects arise from damaged, fibrotic myocardium [4]. Both patchy and diffuse myocardial fibrosis have been described in SSc [2]. Focal fibrosis may be identified with traditional post-contrast cardiac magnetic resonance (CMR) techniques but the advent of T1-mapping sequences and extra-cellular volume quantification (ECV) now enables the characterization of diffuse fibrosis. Abnormalities of T1-mapping are not specific to a single disease but they are a viable biomarker for quantifying the extent of fibrosis, and may potentially indicate reversible changes that can improve with treatment [5]. It is yet to be established whether asymptomatic diffuse fibro-inflammatory myocardial changes in SSc are associated with arrhythmias and whether they may serve as a potential treatment target to reduce the risk of future arrhythmias. We aimed to characterize the burden of fibro-inflammatory cardiac disease in SSc and to evaluate whether fibrotic myocardial disease was associated with arrhythmias.

## Methods

Adult patients (age >18 years) with SSc who fulfilled the 2013 ACR/EULAR SSc classification criteria [6] were invited to participate if they had no history of myocarditis, ischaemic heart disease (based on clinical history and absence of any symptoms), pulmonary arterial hypertension (PAH) diagnosed by right heart catheterization, renal impairment (estimated glomerular filtration rate <40 mL/min/1.73 m^2^), current atrial fibrillation or moderate to severe valvular disease. Patients were deliberately recruited across the spectrum of SSc disease and were eligible for inclusion if they had a disease duration <4 years or >10 years in order to ensure patients with both early and long-standing disease were recruited. Onset of disease was dated from the first presentation of a non-Raynaud’s disease manifestation. All patients were classified according to LeRoy criteria as either lcSSc or dcSSc [7].

Study participants underwent a clinical evaluation and cardiac investigation with CMR and 24-h ambulatory ECG monitoring. CMR data were compared with a group of age- and sex-matched individuals with no history of cardiac disease. All CMR examinations were performed on a 3 T scanner (Magnetom Prisma, Siemens Healthineers, Erlangen, Germany). Patients underwent native T1- and T2-mapping to measure diffuse fibrotic and inflammatory myocardial changes, respectively. Post-contrast imaging was obtained after administration of gadolinium enhancement to visualize any focal fibrotic myocardial changes. CMR was performed and images evaluated according to validated local institutional protocols [8] by two authors (B.C., A.L.G.) blinded to clinical data. A detailed description of the CMR protocol can be found in the Supplementary Materials, available at *Rheumatology* online.

### Ambulatory electrocardiography

All patients underwent ambulatory ECG monitoring with a 3-lead, 24-h Holter monitor (PocketECG, Medicalgorithmics S.A., Warsaw, Poland). Complete traces were assessed for the presence of atrial and ventricular arrhythmias. The number of atrial and ventricular ectopic beats was recorded. Ventricular arrhythmias of interest were ventricular couplets or triplets, ventricular bigeminy, ventricular trigeminy and ventricular tachycardia. Ambulatory ECG data were analysed by one investigator (A.L.G.), blinded to clinical and CMR data.

All patients gave written informed consent to participate in the study. Ethics approval was granted by St Vincent’s Hospital, Melbourne Human Research Ethics Committee (HREC181/18) and the study was performed in accordance with the Declaration of Helsinki. Study data were collected and managed using the REDCap electronic data capture tool hosted at The University of Melbourne.

### Statistical analyses

Continuous variables are presented as mean (s.d.) or median (inter quartile range). Categorical variables are presented as number (percentage). The paired sample *t* test or the Wilcoxon matched-pairs sign-rank test was used to compare continuous variables, as appropriate. The χ^2^ test was used to compare dichotomous data. The relationship between rhythm abnormalities and myocardial fibrosis was evaluated using linear regression analysis for continuous outcome variables and logistic regression analysis for categorical outcome variables. A *p*-value of ≤0.05 was considered statistically significant. Statistical analysis was performed using STATA 15.1 (StataCorp, College Station, TX, USA).

## Results

### Patient demographics

Thirty-four patients were recruited to the study. Thirty-one scans were available for analysis. One patient was unable to tolerate CMR due to claustrophobia and another patient was lost to follow-up prior to cardiac investigations being performed. Images from one CMR were not interpretable due to a high burden of ventricular ectopic beats (VEs). One patient did not receive gadolinium contrast. Baseline characteristics of the study population are presented in Table 1.

**Table 1:** Population characteristics

Demographics	*n* = 31
Age, mean (s.d.), years	55.10 (7.54)
Female, *n* (%)	23 (74)
Disease duration, mean (s.d.), years	9.71 (7.33)
Disease onset <4 years, *n* (%)	12 (39)
lcSSC/dcSSc, *n* (%)	13 (42)/18 (58)
Antibodies, *n* (%)	
Centromere	8 (26)
Scl-70	12 (39)
RNA polymerase III	3 (10)
Cardiac biomarkers, median (IQR)	
Troponin	4 (2, 8)
BNP	31 (13, 52)
Current smokers, *n* (%)	2 (6)
BMI (kg/m^2^), mean (s.d.)	25.00 (4.57)
Hypertension, *n* (%)	2 (6)
Diabetes mellitus, *n* (%)	1 (3)
SSc disease manifestations	
Raynaud phenomenon, *n* (%)	31 (100)
Digital ulcers, *n* (%)	16 (52)
mRSS, median (IQR)	10 (3, 17)
Interstitial lung disease^a^, *n* (%)	10 (32)
FVC (% predicted), mean (s.d.)	88.03 (20.53)
Inflammatory arthritis, *n* (%)	19 (61)
Myositis, *n* (%)	2 (6)
Electrocardiography results	
Average heart rate, mean (s.d.)	79 (8)
Significant heart block or sinus pause >2 s, *n* (%)	0 (0)
Atrial ectopic beats, *n* (%)	28 (90.32)
Number of atrial ectopic beats, mean (s.d.)	1430 (6185)
>50 atrial ectopic beats, *n* (%)	14 (45)
>1000 atrial ectopic beats, *n* (%)	5 (16)
Supraventricular runs, ≥5 beats, *n* (%)	9 (29)
Atrial fibrillation, *n* (%)	1 (3)
Ventricular ectopic beats, *n* (%)	17 (54.84)
Ventricular ectopic beats, mean (s.d.)	197 (527)
>1000 ventricular ectopic beats	2 (6.5)
Ventricular couplets, *n* (%)	5 (16)
Ventricular triplet, *n* (%)	1 (3)
Ventricular bigeminy, *n* (%)	6 (19.35)
Ventricular trigeminy, *n* (%)	3 (9.68)
Non-sustained ventricular tachycardia, *n* (%)	1 (3)
Sustained ventricular tachycardia, *n* (%)	0 (0)

a Any interstitial lung disease detected on high-resolution CT.

BNP: B-type natriuretic peptide; FVC: forced vital capacity; IQR: interquartile range; mRSS: modified Rodnan skin score; Scl-70: anti-topoisomerase.

### Cardiac magnetic resonance imaging findings

SSc patients had a significantly higher LVEF compared with controls (64.38% *vs* 61.09%, p= 0.035) but decreased global longitudinal strain (–16.68% *vs* –20.30%, *p* < 0.001). SSc patients had increased myocardial fibrosis indicated by elevated native T1-times. Twenty-nine (93.5%) patients recorded a native T1-time above the upper limit of normal. Nine patients (30%) had focal regions of late gadolinium enhancement (LGE) detected. SSc patients had increased myocardial oedema measured by T2-mapping time (42.20 *vs* 36.26 ms, *p* < 0.001) (see Supplementary Materials, available at *Rheumatology* online). No SSc disease features were significantly associated with diffuse fibro-inflammatory myocardial disease, including disease duration and extent of cutaneous involvement.

### Ambulatory electrocardiography findings

The 24-h ambulatory ECG findings are summarized in Table 1. One patient recorded an episode of non-sustained ventricular tachycardia (VT) of 4 beats at a rate of 128 beats per min. Asymptomatic paroxysmal atrial fibrillation was detected in one patient, who recorded a 5-h period of rate-controlled atrial fibrillation. There was no association between myocardial fibrosis, measured by native T1 or LGE and the presence of atrial or ventricular ectopy (see Table 2). The one patient who recorded a run of non-sustained VT had no LGE detected on CMR. Ventricular arrhythmias (couplets, triplets, bigeminy, trigeminy, VT) were not associated with either elevated T1-mapping or T2-mapping times or the presence of LGE.

**Table 2:** Comparison of CMR parameters and electrocardiography abnormalities

	Ventricular ectopic beats			Atrial ectopic beats		
	Coef.	*P*-value (95% CI)	R^2^	Coef.	*P*-value (95% CI)	R^2^
Native T1-time (SASHA)	0.38	0.858 (−3.94, 4.71)	0.001	3.23	0.898 (−47.56, 54.02)	<0.001
Native T1-time (ShMOLLI)	3.83	0.125 (−1.13, 8.79)	0.079	53.96	0.063 (−3.15, 111.08)	0.114
Native T2-time	−45.80	0.074 (−96.37, 4.77)	0.106	−200.79	0.515 (−823.79, 422.20)	0.015
LGE	124.59	0.568 (−316.95, 566.12)	0.012	3461.83	0.171 (−1583.55, 8507.20)	0.066

CMR: cardiac magnetic resonance imaging; ECV: extra-cellular volume; LGE: late gadolinium enhancement; SASHA: saturated recovery single-shot acquisition; ShMOLLI: shortened Modified look locker sequence.

## Discussion

In a study of patients with SSc and no clinical evidence of cardiac disease we found that both myocardial fibro-inflammation on CMR and arrhythmias were highly prevalent. However, there was no clear association between these two abnormalities.

Diffuse myocardial fibrosis was clearly present, as evidenced by differences in the group means for T1-mapping values and the fact that almost every patient with SSc had values above established normal reference values [8]. Unexpectedly, we detected pathological myocardial fibrosis in patients early in their disease course with limited skin involvement and no significant internal organ involvement, a patient group generally expected to have a more benign disease course. Our results show that there are no reliable clinical indicators to predict which patients are at higher risk of diffuse myocardial fibrosis.

Our results concur with previous studies that have shown diffuse myocardial fibrosis is a characteristic hallmark of SSc [9–15]. However, the exact prognostic implications of myocardial fibrosis in the absence of cardiac symptoms remain unclear. The characteristic fibrosis associated with SSc is known to involve the myocardium with the potential to manifest as arrhythmias or sudden death. In other cardiomyopathies, myocardial fibrosis serves as a potential substrate for arrhythmias [16] and ventricular ectopy in structurally abnormal hearts is linked to an increased risk of death [17]. Higher ECV has been associated with an increased risk of major cardiovascular events and mortality in non-ischaemic cardiomyopathy, valvular heart disease and amyloidosis [16].

Although we did not find an association between greater ECV and arrhythmias, a potential for clinical events resulting from diffuse myocardial disease in SSc remains. Prior studies have evaluated the potential link between myocardial fibrosis and arrhythmias. A recent study that included participants with cardiac symptoms or events proposed that a combination of myocardial oedema measured by T2 ratio and percentage burden of LGE could be used to predict the development of rhythm disturbances [18]. A prospective study that included evaluation with T1-mapping sequences demonstrated an increased risk of cardiac events, including rhythm disturbances, in patients with high native T1-times [15]. However, this study included patients with PAH, a history of renal crisis and ischaemic heart disease, all of which may potentially confound results. It has been hypothesized that myocardial fibrosis in SSc accrues as a result of microcirculatory dysfunction, a so-called ‘cardiac RP’ [19]. Myocardial microvascular dysfunction, particularly in those patients with other vascular manifestations such as PAH or renal crisis, may in part explain the burden of diffuse fibrosis observed in these patients.

This study deliberately selected participants to exclude important cardiovascular comorbidities such as PAH and ischaemic heart disease that commonly confound cardiac assessment of SSc patients. Our observed absence of an association between fibrosis and arrhythmias is consistent with a study by Bissell *et al.* which assessed patients with no ostensible cardiovascular disease using an implantable loop recorder over 3 years. They observed a high prevalence of arrhythmias but no significant association with CMR parameters of fibrosis [20]. It is likely the prognostic significance of CMR evidence of myocardial fibrosis needs to be considered in light of the presence of cardiac symptoms and its potential utility in the prediction of poor outcomes is in those patients who report cardiac symptoms.

There is significant interest in the early detection of SHI during an active, potentially reversible phase of myocardial disease as a strategy to improve patient outcomes. However, our results suggest that, at least in the early stages of SSc, structural heart disease and electrophysiological abnormalities do not develop or progress in parallel and that a complete cardiac evaluation requires both structural and functional studies as well as interrogation for any electrophysiological changes. Whether reversal of structural heart muscle changes can limit the development of future arrhythmias or reduce the risk of sudden cardiac death remains unknown.

A limitation of this study was the lack of ECG control data. VEs are a common finding in the general population and a higher burden of VEs has been associated with poorer cardiac outcomes and increased mortality [17]. While we acknowledge that this study may have been underpowered to detect an association between fibrosis and arrhythmias, this study serves as an important counterbalance to studies of similar size that have proposed positive associations despite the risk of Type I error. The lack of association, even though myocardial fibrosis and arrhythmias were relatively common in this study, suggests that caution is required in basing clinical decisions on these non-invasive surrogates. It is possible that with more extended monitoring a higher frequency of arrhythmic events would have been recorded. The yield of prolonged ECG monitoring in SSc is unknown. More prolonged monitoring usually necessitates the implantation of a monitoring device, the indications for which are ill-defined in SSc, particularly given concerns about wound healing in patients with significant cutaneous chest wall involvement and infection risk for immunosuppressed patients.

It is possible that the clinical significance of myocardial fibrosis in SSc only becomes apparent over time and this study was limited by a lack of clinical follow-up, meaning our results may underestimate the relationship between myocardial fibrosis and arrhythmia. We did not have any histopathological validation of the CMR findings; however, it would have been unethical to include biopsy as part of this study given the invasive nature of the procedure and its inherent risks.

We have demonstrated that diffuse myocardial fibrosis is a near-universal finding among SSc patients without cardiac symptoms or history of SHI, regardless of disease duration or extent of skin involvement, and that it can be considered a hallmark of the disease. CMR has an emerging role in the diagnosis of SHI, however its prognostic utility remains unproven. We observed a high prevalence of important abnormalities using electrocardiography and CMR, however we found no clear association between cardiac arrhythmias and the extent of myocardial fibrosis in patients without clinical symptoms of cardiac disease. We reinforce evidence that SSc patients are at increased risk of cardiac complications but simultaneously temper enthusiasm for the use of non-invasive markers of myocardial fibrosis in assessing risk of significant ventricular arrhythmia in the absence of cardiac symptoms and in those patients without clinical suspicion of SHI.

*Funding*: This work was supported by a National Heart Foundation of Australia Vanguard Grant [102206], Arthritis Australia—Australian Rheumatology Association Project Grant, Scleroderma Victoria Project Grant, St Vincent’s Hospital Research Endowment Fund Project Grant [88371], The University of Melbourne Research Establishment Grant, *MSK Australia* PhD Scholarship and an Australian Government Research Training Program Scholarship to L.R., the University of Melbourne Research Training Scholarship to Z.B., National Health and Medical Research Council of Australia Investigator Grant [GNT 1176538] to M.N. and the Australian National Heart Foundation Future Leader Fellowship [102021] to A.L.G.

*Disclosure statement*: M.N. receives research support from Actelion, AstraZeneca, Bristol Myers Squibb, GlaxoSmithKline, Janssen and UCB. M.N. has received honoraria from Actelion, Boehringer Ingelheim, Eli Lilly, GlaxoSmithKline, Janssen, Pfizer and UCB. All other authors have no conflicts of interest to disclose. *Data availability statement*: The data underlying this article will be shared on reasonable request to the corresponding author.

## Supplementary Material

keac065_Supplementary_Data

## Data Availability

The data underlying this article cannot be shared publicly due for the privacy of the individuals that participated in the study. The data will be shared on reasonable request to the corresponding author.
