# Supplementary material for: Myocardial fibrosis and arrhythmic burden in systemic sclerosis
Source: Rheumatology (Oxford). 2022 Feb 8;61(11):4497–502. doi: 10.1093/rheumatology/keac065 (PMC9629381; doi:10.1093/rheumatology/keac065)
Supplement: keac065_Supplementary_Data [file keac065_supplementary_data.docx]

**Supplementary materials**

*Cardiac magnetic resonance imaging protocol*

All CMR examinations were performed on a 3T scanner (Magnetom Prisma, Siemens Healthineers, Erlangen, Germany). All image post-processing was performed using a dedicated software package (CVI42, Circle Cardiovascular Imaging, Calgary, Canada).

Three long-axis and a contiguous short-axis stack of cine images (8-mm slice thickness, no gap) were acquired using an ECG-gated balanced steady-state free precision sequence in expiration. Left ventricular (LV) mass, end-diastolic volume, end-systolic volume, and LV ejection fraction (LVEF) were quantified using CVI42 using a summation of disk method. Papillary muscles were regarded as part of the ventricular cavity. Measurements were indexed to body surface area.

Myocardial T1-times were derived using the saturated recovery single-shot acquisition (SASHA) and shortened modified look locker (ShMOLLI) sequences which automatically generated pixel maps of T1-times. These were used during post-processing with a motion correction algorithm applied to the raw images. Post-contrast sequences were analysed 10-minutes following intravenous bolus injection of gadolinium-diethylene triamine penta-acetic acid (0.2 mmol/kg BW, Magnevist, Schering, Germany).

Each sequence was acquired within an end-expiration breath-hold using an ECG-triggered single-shot acquisition with a balanced steady-state free precession readout in a single mid-LV short-axis slice. Native T1 (pre-contrast) and post-contrast T1-times were measured in the myocardium and left ventricular blood pool using a region of interest on the T1 pixel map. T1 measurements were taken at the mid SAX level, both by including the entire myocardium and by taking a region within the septum.[1, 2] The ECV was calculated from the dynamic steady state concentration of extracellular contrast in the myocardium relative to the blood pool, incorporating the participant’s haematocrit, as previously described.[1, 3]

Myocardial T2-times were measured using a T2-prepared fast angle low shot (FLASH) sequence that acquires three single-shot T2-weighted images in the same diastolic phase, each with a different T2 preparation time (preparation times 0ms, 25ms, 55ms). A non-rigid image registration algorithm is used for in-plane motion correction before subsequent pixel-wise fitting of a two-parameter equation assuming a mono-exponential T2 signal decay. Typical imaging parameters for each of these techniques are detailed in Table 1.

*Table 1: Typical imaging parameters for CMR sequences*

|  | **SSFP** | **LGE** | **SASHA** | **ShMOLLI** | **T2 prepared FLASH** | **T2*** |
| --- | --- | --- | --- | --- | --- | --- |
| Repetition time (TR) | 38.4 | 835.2 | 733.08 ms | 378.6 ms | 214.94 ms | 700 ms |
| Echo Time (TE) | 1.41 ms | 1.55ms | 1.26ms | 1.07 ms | ~1.12 ms | ~2.27ms |
| Phases | 25 | -- | -- | -- | -- |  |
| Slice thickness | 8.0 mm | 8.0 mm | 8.0 mm | 8.0mm | 8.0mm | 8.0mm |
| Inversion time |  | Per TI scout  [250-350ms] | ~600 ms [multiple saturation times] | ~260 ms [multiple inversion times] | -- | -- |
| Field of view read | 380 mm | 350 mm | 360 ms | 360 mm | 360 mm | 380mm |
| Voxel size (mm) | 1.7x1.7x 8.0 | 1.4x1.4x8.0 | 1.4x1.4x8.0 | 0.9x0.9x8.0 | 0.9x0.9x8.0 | 1.5x1.5x 8.0 |
| Flip angle (^o^) | -- | 20 | 50 | 35 | -- | -- |
| Bandwidth (Hz/Pixel) | 970 | 465 | 1085 | 898 |  |  |

*Abbreviations:* CMR: cardiac magnetic resonance imaging; FLASH: fast angle low shot; LGE: late gadolinium enhancement; SASHA: saturated recovery single-shot acquisition; ShMOLLI: shortened modified look locker; SSFP: steady-state free precision

*Evaluation of images*

Regional myocardial fibrosis was visually identified by delayed enhancement within the myocardium, defined as areas of increased signal intensity post-contrast when compared to nulled healthy myocardium. T1 and T2 measurements were taken within a region of the septum. Any mid-wall fibrosis (typical of dilated cardiomyopathy) was included, with the consideration that this represents a continuum with diffuse interstitial fibrosis.[4]

*Evaluation of global longitudinal strain*

Global longitudinal strain (GLS) was calculated using feature tracking on the cine images on CVI42 software. The myocardium was defined according to American Heart Association segments by placing a marker across the mitral valve annulus and from the annulus to the apex on long axis images, and by marking endocardial and epicardial borders in the short axis volumetric stack and three apical cine images (4 chamber, 2 chamber, 3 chamber). Markers were placed at both right ventricle (RV) insertion points on the short axis images. The feature-tracking algorithm within the CVI42 software calculated GLS.

*Table 2: Comparison of T1-mapping between systemic sclerosis patients and healthy controls*

|  | **n (per group)** | **SSc patients** | **Control subjects** | **p value** |
| --- | --- | --- | --- | --- |
| **Demographics** | | | | |
| Female (n,%) | 31 | 23 (74%) | 23 (74%) | 1.00 |
| Age (years) (mean, SD) | 31 | 55.10 (7.54) | 56.32 (9.66) | 0.027 |
| Weight (kg) (mean, SD) | 31 | 68.08 (13.14) | 69.69 (14.73) | 0.554 |
| **CMR parameters** |  |  |  |  |
| LVEF (%) (mean, SD) | 31 | 64.38 (6.37) | 61.09 (5.06) | 0.035 |
| LV global longitudinal strain (%) (mean, SD) | 28 | -16.68 (2.31) | -20.30 (3.41) | <0.001 |
| LV end diastolic volume (mL) (median, IQR) | 31 | 119  (106-147) | 149.3  (121-174.9) | <0.001 |
| LV myocardial mass (g)  (median, IQR) | 31 | 100  (86-115) | 101.4  (77.3 – 119) | 0.394 |
| RVEF (%) (mean, SD) | 31 | 51.33 (3.06) | 47 (1) | 0.186 |
| RV end diastolic volume (mL) (mean, SD) | 31 | 162.33  (31.37) | 196 (26) | 0.151 |
| Late gadolinium enhancement present (n,%) | 30 | 9 (30%) | 0 (0%) | <0.001 |
| T2-mapping time (ms) (mean, SD) | 27 | 42.20 (3.83) | 36.26 (1.78) | <0.001 |
| *SASHA* | | | | |
| Native T1-time (ms) (mean, SD) | 31 | 1583.81 (46.24) | 1515.29 (36.43) | <0.001 |
| ECV (%) (mean, SD) | 28 | 25 (2) | 21 (2) | <0.001 |
| *ShMOLLI* | | | | |
| Native T1-time (ms) (mean, SD) | 31 | 1218.47 (38.71) | 1138.06 (37.74) | <0.001 |
| ECV (%) (mean, SD) | 29 | 31 (3) | 26 (3) | <0.001 |

*Abbreviations:* ECV: extra-cellular volume; IQR: interquartile range; LV: left ventricle; LVEF: left ventricular ejection fraction; RV: right ventricle; RVEF: right ventricular ejection fraction; SASHA: saturated recovery single-shot acquisition; SD: standard deviation; ShMOLLI: shortened Modified look locker sequence

**References**

1 Messroghli DR, Moon JC, Ferreira VM, Grosse-Wortmann L, He T, Kellman P et al. Clinical recommendations for cardiovascular magnetic resonance mapping of T1, T2, T2* and extracellular volume: A consensus statement by the Society for Cardiovascular Magnetic Resonance (SCMR) endorsed by the European Association for Cardiovascular Imaging (EACVI). J Cardiovasc Magn Reson 2017;19:75.

2 Iles L, Pfluger H, Phrommintikul A, Cherayath J, Aksit, P, Gupta SN et al. Evaluation of diffuse myocardial fibrosis in heart failure with cardiac magnetic resonance contrast-enhanced T1 mapping. J Am Coll Cardiol 2008;52(19):1574-80.

3 Kellman P, Wilson JR, Xue H, Ugander M, Arai AE. Extracellular volume fraction mapping in the myocardium, part 1: evaluation of an automated method. J Cardiovasc Magn Reson 2012;14:63.

4 Miller CA, Naish JH, Bishop P, Coutts G, Clark D, Zhao S et al. Comprehensive validation of cardiovascular magnetic resonance techniques for the assessment of myocardial extracellular volume. Circ Cardiovasc Imaging 2013;6(3):373-83.
